# The Development of a Flexible Bodily Representation: Behavioral Outcomes and Brain Oscillatory Activity During the Rubber Hand Illusion in Preterm and Full-Term School-Age Children

**DOI:** 10.3389/fnhum.2021.702449

**Published:** 2021-09-14

**Authors:** Letizia Della Longa, Giovanni Mento, Teresa Farroni

**Affiliations:** ^1^Developmental Psychology and Socialization Department, University of Padua, Padua, Italy; ^2^General Psychology Department, University of Padua, Padua, Italy; ^3^PNC Padua Neuroscience Centre, University of Padua, Padua, Italy

**Keywords:** body ownership, multisensory integration, rubber hand illusion, development, preterm children, oscillatory activity

## Abstract

During childhood, the body undergoes rapid changes suggesting the need to constantly update body representation based on the integration of multisensory signals. Sensory experiences in critical periods of early development may have a significant impact on the neurobiological mechanisms underpinning the development of the sense of one’s own body. Specifically, preterm children are at risk for sensory processing difficulties, which may lead to specific vulnerability in binding together sensory information in order to modulate the representation of the bodily self. The present study aims to investigate the malleability of body ownership in preterm (*N* = 21) and full-term (*N* = 19) school-age children, as reflected by sensitivity to the Rubber Hand Illusion. The results revealed that multisensory processes underlying the ability to identify a rubber hand as being part of one’s own body are already established in childhood, as indicated by a higher subjective feeling of embodiment over the rubber hand during synchronous visual-tactile stimulation. Notably, the effect of visual-tactile synchrony was related to the suppression of the alpha band oscillations over frontal, central, and parietal scalp regions, possibly indicating a greater activation of somatosensory and associative areas underpinning the illusory body ownership. Moreover, an interaction effect between visual-tactile condition and group emerged, suggesting that preterm children showed a greater suppression of alpha oscillatory activity during the illusion. This result together with lower scores of subjective embodiment over the rubber hand reported by preterm children indicate that preterm birth may affect the development of the flexible representation of the body. These findings provide an essential contribution to better understand the processes of identification and differentiation of the bodily self from the external environment, in both full-term and preterm children, paving the way for a multisensory and embodied approach to the investigation of social and cognitive development.

## Introduction

The sense of body ownership, which is the feeling that our body belongs to ourselves, represents a central component of the developing sense of bodily self-awareness and the process of differentiation of the self from the others (Tsakiris, 2016). This implicit knowledge about our own body allows us to correctly identify and localize ourselves in the complexity of a multisensory environment, providing a reference for all experiences from the external world, thus it can be considered a prerequisite for the development of perceptual, cognitive, and social abilities. Normally, we take the experience of our body for granted, however the ability to perceive and recognize our body is a result of complex integration processes of signals coming from different sensory modalities (Ehrsson, 2012).

The ability to perceive spatio-temporal synchrony through the body lies at the core of the development of bodily self-awareness from infancy onwards. From a developmental perspective, bodily self-awareness emerges in parallel to the acquisition of somatosensory skills as infants develop an implicit knowledge of themselves by interacting with the surrounding environment (Riva, 2018). Sensorimotor exploration not only provides information about the external world but also includes the feeling of being the subject of a given experience, establishing a foundation for self-awareness. Empirical observations suggest that, from the first days of life, newborns already show an implicit sense of bodily self-awareness based on the integration of different sensory information (Rochat and Hespos, 1997; Rochat and Striano, 2000). This early ability to differentiate sensations originating from within or outside the body represents the most basic self-experience (Rochat, 2003), providing a foundation for the development of self-other interactions. Moreover, from 2-month of age infants start to systematically explore their own body and the perceptual consequences of self-produced actions, developing a sense of the bodily self as differentiated, situated, and agent in the environment (Rochat and Striano, 2000). Developmental studies have indeed demonstrated that infants differentiate sensations originating from within and outside the body, by showing the ability to discriminate visual-proprioceptive (Bahrick and Watson, 1985; Rochat and Morgan, 1995; Morgan and Rochat, 1997), visual-tactile (Zmyj et al., 2011; Filippetti et al., 2013, 2016; Della Longa et al., 2020) and visual-interoceptive contingencies (Maister et al., 2017). This suggests that implicit bodily self-awareness is based on multisensory integration of bodily signals and early detection of synchrony between vision and sensory feedback from the body.

The early ability to detect visual-tactile body-related contingencies paves the way to a protracted process of self-awareness. During the first years of life other abilities gradually emerges, such as mirror self-recognition (children begin to match their own facial and body movements with the image of themselves in a mirror), self-referential language (personal pronouns usage), emotions related to social contexts (embarrassment, guilt, pride; Lewis, 2011), suggesting toddlers begin to perceive themselves acting and interacting in the surrounding physical and social environment. The emergence of the representation of the body as an object in relation to other objects together with the sense of ownership over the body represents a central component of the developing bodily self-awareness, which underpins the acquisition of motor and socio-cognitive abilities. During childhood multisensory processes underlying the sense of body ownership gradually develop supporting children’s ability to update the representation of a body that rapidly changes and grows. To the end of investigating the plasticity of body ownership, body illusions have been used in both adult and developmental populations.

The rubber hand illusion (RHI; Botvinick and Cohen, 1998) is a well-established paradigm to investigate the formation and modulation of the sense of body ownership based on the integration of multisensory information. In a typical RHI paradigm, the participant sees a rubber hand that lies in an anatomical plausible position, while the real hand is covered. The rubber hand, as well as the participant’s own hand, are stroked synchronously, creating the multisensory conflict of seeing a touch that is felt at a different location. This multisensory conflict is resolved by incorporating the rubber hand in one’s own body representation (subjective embodiment), as well as by relocating the perceived position of one’s own hand towards the rubber hand (proprioceptive drift). These two correlates of the RHI reflect complementary mechanisms of body perception: the feeling of embodiment reflects the experience of body ownership, while the proprioceptive drift is related to the location of the body in space (Serino et al., 2013). The illusion indicates that body representation is continuously updated from sensory input and it can be modulated to include external objects.

Only a few studies have investigated the development of susceptibility to the RHI across childhood, showing preliminary evidence that children are able to flexibly modulate their body representation according to contingent visual-tactile input (Lee et al., 2021). All these studies consistently reported that children showed a stronger feeling of embodiment over the rubber hand during synchronous visual-tactile stimulation compared to the asynchronous condition, suggesting an early developing visual-tactile process underpinning the sense of body ownership (Cowie et al., 2013, 2016; Nava et al., 2017; Filippetti and Crucinelli, 2019). Importantly, the subjective experience induced during the illusion seems to be stable across different ages. By contrast, the findings regarding the perceived hand position in children are more complex. While some studies reported a greater proprioceptive recalibration towards the rubber hand in younger children, possibly indicating a strong reliance on visual information (Cowie et al., 2016), other findings failed to evidence the same developmental pathway (Nava et al., 2017; Filippetti and Crucinelli, 2019). Despite the fact that different methodological approaches make it difficult to compare results across studies (Lee et al., 2021), preliminary evidence suggests that the RHI has different psychological and physiological effects, which are experimentally dissociable and develop at different rates during childhood (Rohde et al., 2011; Abdulkarim and Ehrsson, 2016; Cowie et al., 2016). Further research is needed to better understand how multisensory integration processes underpinning body representations gradually develop. In particular, the neural mechanisms underpinning the processes of self-other differentiation are poorly understood from a developmental perspective. To our knowledge, no developmental studies have specifically investigated brain activity supporting the experience of body ownership during the RHI.

Considering the brain mechanisms underpinning the RHI, adult studies investigated somatosensory evoked potentials (SEP) showing an enhancement of late SEP components following synchronous tactile stimulation, which may reflect activation of premotor and parietal cortices (Press et al., 2008). These results are further supported by functional neuroimaging studies that evidenced a network of brain areas involved in the experience of body ownership, including the premotor cortex, sensorimotor cortex, intraparietal sulcus, temporoparietal junction, and insula (Ehrsson et al., 2004, 2005; Tsakiris et al., 2007; Tsakiris, 2010; Blanke, 2012). Moreover, studies of neural oscillation have also started to contribute to the understanding of the neural dynamics related to own-body perception. Neural oscillations in different frequency bands reflect separate mechanisms of multisensory processing, including local neural oscillations and functional connectivity between distant cortical areas (Keil and Senkowski, 2018). More specifically, bottom-up processing has been shown to engage local networks in high-frequency bands (>30 Hz), whereas top-down control through long-range integrative processing engages low-frequency bands (<30 Hz; Keil and Senkowski, 2018). In the context of the RHI, an increase of interelectrode phase synchrony in the gamma-band frequency has been evidenced over parietal regions, signaling crossmodal integration of visual and tactile signals during the induction of the illusion (Kanayama et al., 2007, 2009). Analysis of oscillatory activity also revealed that the emergence of the illusory sense of ownership over the rubber hand was related to modulation of oscillatory power in the alpha band during the RHI (Evans and Blanke, 2013; Rao and Kayser, 2017) as well as in the full body illusion (Lenggenhager et al., 2011) and in the somatic RHI (Faivre et al., 2017). These studies evidenced a relative decrease in alpha power over frontoparietal regions during the illusion, which is not associated with visual information or specific control condition, as it emerged from a combination of contrasts (spatial congruency/incongruency and temporal synchrony/asynchrony; Rao and Kayser, 2017), suggesting that modulation of alpha activity can be considered an important neurophysiological marker of body ownership during the induction of the RHI.

Therefore, the first objective of the present study is to explore EEG oscillatory activity related to visual-tactile integration processes that may represent the neurophysiological basis for the development of the sense of bodily ownership during childhood. To achieve this purpose, we use the RHI paradigm focusing on the classical behavioral measures (proprioceptive drift and subjective embodiment) as well as on the neural oscillatory activity, which may support the integration of multisensory signals in order to modulate body representation accordingly to the concurrent sensory input. More specifically, we decided to measure oscillatory activity during continuous visual-tactile stimulation, comparing the synchronous condition, which should induce the illusion, and the asynchronous condition, which prevents the integration of conflicting visual and tactile input. In contrast to event-related paradigms, continuous recording paradigms do not rely primarily on temporally defined events, thus they discard temporal information and focus instead on spectral information and their experimental induced changes (Gross, 2014). We hypothesized that synchronous visual-tactile stimulation would induce an increased activity in multisensory related brain circuits resulting in desynchronization of alpha oscillatory activity and thus a decrease of the power spectrum density.

A second purpose of the present study is to investigate possible differences in the modulation of body representation in full-term and preterm children. To our knowledge, no studies have specifically addressed the development of self-other differentiation and bodily awareness in children with multisensory processing vulnerability, such as children born preterm. Preterm birth is defined as a birth occurring before the 38th week of gestation and it is associated with increased risk for early brain damage due to hypoxia-ischemia and inflammation affecting in particular the cerebral white matter (Aarnoudse-Moens et al., 2009; Volpe, 2009). Moreover, detrimental environmental factors of the neonatal intensive care (NICU) may increase the vulnerability to develop neurodevelopmental difficulties (Anand and Scalzo, 2000; Mento and Bisiacchi, 2012). In NICU the pattern of sensory stimulation is radically altered, exposing preterm newborns to a stressful environment, which they are not developmentally prepared to handle. On one side, preterm newborns are presented with sensory overstimulation due to bright lights, noise, nursery handling, repetitive painful procedures (e.g., heel lancing, venipunctures, nasal suctioning). On the other side, preterm newborns suffer from sensory deprivation in terms of parental affective care (tactile, vestibular, and kinesthetic stimulation; Nair et al., 2003; Machado et al., 2017). This leads preterm infants to an increased risk for sensory processing dysfunctions, including the ability to integrate multisensory information (Mitchell et al., 2014; Machado et al., 2017). Sensory difficulties have been shown to persist in preterm school-age children affecting somatosensory and motor processing (Niutanen et al., 2020) with important implications for neurocognitive and behavioral outcomes (Bröring et al., 2017; Ryckman et al., 2017). However, multisensory integration and body processing in preterm children have not received much attention, with only a few studies suggesting that in infancy preterm children showed poor visual-tactile integration, atypical reactivity to tactile and vestibular stimulation (Bart et al., 2011; Lecuona et al., 2016) and reduced sensorimotor control (Delafield-Butt et al., 2018), which reflect a prerequisite for the emergence of an implicit sense of bodily self-awareness. Moreover, a recent study evidenced that preterm children showed difficulties in the visual perceptual processing of body representation as reflected by visuospatial judgments on body stimuli (Butti et al., 2020). This finding points to possible long-lasting consequences of preterm birth in children’s ability to integrate multisensory information to create a representation of their body coherent with sensory input. For this reason, we proposed to include a group of children born preterm in order to examine possible differences in the susceptibility to the RHI between children born preterm and full-term. Considering that preterm birth is frequently associated with increased vulnerability for cerebral white matter damage (Volpe, 2009) together with inadequate early sensory experiences (Grubb and Thompson, 2004), it is plausible that preterm children might present difficulties in the ability to integrate multisensory bodily signals in order to develop a coherent and flexible representation of the bodily self. Thus, we hypothesized that preterm children would show atypical modulation of the sense of body ownership. More specifically, we expected that preterm children would be less sensitive to the RHI compared to children born full-term, showing difficulties in adapting their body representation to the available multisensory information.

## Materials and Methods

### Participants

The study was conducted at the Department of Developmental Psychology and Socialization of the University of Padua. Forty children between the ages of 6 and 11 years old were included in the study (21 children born preterm and 19 children born full-term). Participants in the preterm group were recruited from the association “Pulcino” in Padua, a center for children born preterm that provides support for premature infants and their families from the earliest stages of development since later childhood. Participants in the control group were recruited from the local community. Participants’ characteristics are summarized in Table 1. Parents gave written consent for their child’s participation after being informed about the whole procedure. The local Ethical Committee of Psychological Research (University of Padua) approved the study protocol.

**Table 1:** Sample characteristics.

	Preterm children	Full-term children
N (%male)	21 (42.86%)	19 (31.58%)
Age (months)	103.9 (17.0); range 78–136	104.1 (15.2); range 81–139
Gestational age (weeks)	30.0 (3.27); range 24–36 Mild preterm (32–36 weeks) *N* = 8 Very preterm (28–31 weeks) *N* = 8 Extremely preterm (<28 weeks) *N* = 5	All >38
Birth weight (gram)	1,443.76 (622.69); range 512–2,500	All >2,500

### Stimuli and Procedure

In order to ensure comprehension of task instructions and comparable cognitive abilities between the two groups, each participant was asked to complete a cognitive assessment in the first session, and then he/she was presented with the RHI paradigm during high-density EEG (hdEEG) recording in a second session. We opted to carry out the cognitive assessment and the experimental task/hdEEG recording in two separate sessions in order to reduce testing time for children, thereby ensuring optimal performance.

In the first session, each participant was asked to completed the Raven’s Colored Progressive Matrices (CPM; Raven, 1958) to evaluate abstract reasoning, the digit span test forward and backward (BVN 5–11; Bisiacchi et al., 2005) to estimate the working memory span, the Attention Network Task (ANT; Rueda et al., 2004), which provides a measure of three main components of attention (alerting, orienting, and executive control), and a computerized version of the Berg Card Sorting Test (BCST; Berg, 1948) for assessing cognitive flexibility. Moreover, parents were asked to fill some questionnaires to investigate the sensory, cognitive, emotional and behavioral functioning of children in everyday activities. Specifically, we included the Strengths and Difficulties Questionnaire (SDQ; Marzocchi et al., 2002), which investigate the presence of behavioral and emotional difficulties as well as prosocial behaviousr; the Emotion Regulation Checklist (ERC; Shields and Cicchetti, 1997; Molina et al., 2014), which investigate negativity and emotion regulation; Temperament in Middle Childhood Questionnaire (TMCQ; Simonds and Rothbart, 2004), which investigate the temperament of the child in the last 6 months; Behavioral Rating Inventory of Executive Function (BRIEF; Gioia et al., 2000), which investigates executive functioning. Finally, a purpose-built sensory questionnaire was used to explore children’s sensory skills, focused on somatosensory and body-related processing. This questionnaire was specifically designed for the current research study in order to collect information about different areas of sensory processing involved in daily activities, including discriminative touch, affective touch, interoceptive sensitivity, proprioception, and body awareness.

**Figure 1:**
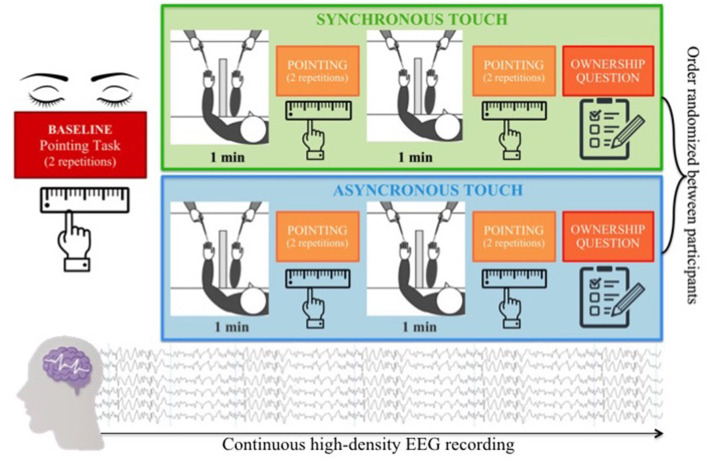
RHI paradigm: each child was presented with two experimental blocks, varying the visual-tactile congruency (synchronous vs. asynchronous). Each block consisted of two 1-min visual-tactile stimulations immediately followed by a pointing task to measure the proprioceptive drift and a final question investigating the subjective feeling of ownership over the rubber hand. High-density EEG data were continuously recorded during the experimental session. RHI, rubber hand illusion.

In the second session, each participant was presented with the RHI paradigm, based on the procedure developed by Botvinick and Cohen (1998). Each child was seated in front of a table directly across from the experimenter. The participant placed his/her left hand on the table and was trained to slide his/her right index finger following a ridge under the table to find the point underneath the left index finger (pointing response task). After training, the participant was asked to repeat twice the pointing response task with eyes closed to estimate his/her baseline ability to localize the position of his/her own hand. Specifically, the baseline estimation error of hand position was calculated as the difference between mean pointing response and the actual hand position. Positive values indicate a shift towards the body midline from the actual hand position, whereas negative values indicate a shift away from the body midline. After that, a panel obstruction was placed on the table to prevent the participant from viewing his/her own hand and a fake rubber hand was placed on the table at a distance of 15 cm on the right from the actual hand position. A black cloth was placed around the child to cover part of both the real and the rubber arm. The trained experimenter stroked the participant’s hand and the rubber hand with two identical brushes either synchronously or asynchronously. During the stimulation, the participant was asked to closely watch the rubber hand. Each participant was presented with two RHI blocks. In each block, the experimenter administered the same tactile stimulation twice (two trials), each time for 1 min. The tactile stimulation was manipulated between blocks, varying the synchrony between the touch on the real hand and the visual feedback on the rubber hand (synchronous—same time and same position- vs. asynchronous—different time and different position). The order of the experimental conditions was randomized between participants. As in the baseline, the same pointing response task was administered following each RHI trial. Therefore, the participant pointed four times for each experimental condition. To measure the extent to which the proprioceptive perceived position of one’s own hand was influenced by incongruent multisensory signals (induction phase of the RHI), proprioceptive drift was calculated by subtracting the mean baseline pointing response from the mean test pointing response for each experimental condition. After each RHI block, the participant was asked two questions, the first one concerned the sense of embodiment felt over the rubber hand “When I was stroking with the brush, did you feel like the rubber hand was your hand?”; the second one was a control question “When I was stroking with the brush, did you feel to have three hands?.” A was designed to be easily understood by children: 1 = no, definitely not; 2 = no; 3 = no, not really; 4 = in between; 5 = yes, a little; 6 = yes, a lot; 7 = yes, lots and lots (Figure 1). Finally, we were interested in exploring the modulation of neural oscillations underpinning bodily illusion, thus we continuously record participants’ hdEEG activity during the experimental session.

### EEG Recording and Processing

During the entire RHI task, hdEEG data were continuously recorded. We used a Geodesic high-density EEG System (EGI GES-300) with a pre-cabled 128-channel Hydrocel Geodesic Sensor Net (HCGSN-128) and electrical reference to the vertex. The sampling rate was 500 Hz and the impedance was kept below 60 kΩ for each sensor. Signal pre-processing was performed through EEGLAB 14.1.2b (Delorme and Makeig, 2004). The continuous EEG signal was first segmented according to experimental conditions (synchronous vs. asynchronous visuotactile stimulation), resulting in two different 2-min experimental blocks for each participant. The EEG data from different recording blocks were pre-processed separately. The signal was downsampled at 250 Hz and then bandpass-filtered (1–40 Hz) using a Hamming windowed sinc finite impulse response filter (filter order 1/4 8250). Manual inspection was done for each subject in order to eliminate those segments of signal that presented huge artifacts amenable to body movements. Successively, data cleaning was performed by means of an independent component analysis (ICA; Stone, 2002) using the algorithm implemented in EEGLAB. The resulting independent components were visually inspected and those clearly related to eye blinks, eye movements, and muscle artifacts were discarded. Channels presenting artifactual activity were eliminated and their activity was reconstructed with spherical interpolation (Perrin et al., 1989; Ferree, 2006). Finally, the data were then re-referenced to the average of all electrodes. At this stage, EEG data were imported in Brainstorm (Tadel et al., 2011) to analyze the individual oscillatory activity for each experimental condition. We applied Welshed power spectrum density to decompose the raw EEG data into distinct frequencies from 1 to 40 Hz using the Brainstorm software. As we were mainly interested in changes of oscillatory activity in the alpha band, we considered the averaged power spectrum density between 8 and 12 Hz. We analyzed changes in this frequency band because alpha power has been associated with bodily self-awareness (Lenggenhager et al., 2011; Evans and Blanke, 2013; Rao and Kayser, 2017). No baseline normalization was performed but within-subject statistical comparisons were used (see below), which makes the subtraction of a common baseline unnecessary (Rao and Kayser, 2017).

### Statistical Analyses

#### Behavioral Statistical Analyses

All statistical analyses were performed using R, a software environment for statistical computing and graphics (R Core Team, 2016). To test for group differences in the performance at the cognitive assessment and in the parent-reported questionnaires, we carried out *t*-tests; while to analyze data from the proprioceptive drift and the embodiment experience we used a mixed-effect model approach. The choice of using a mixed-effects model approach was determined by the possibility to take into account fixed effects, which are parameters associated with an entire population as they are directly controlled by the researcher, and random effects, which are associated with individual experimental units randomly drawn from the population (Gelman and Hill, 2007; Baayen et al., 2008). Akaike information criterion (AIC) model comparison has been used to compare a set of models fitted to the same data (Akaike, 1973; McElreath, 2016). The model that produces the lowest AIC value is the most plausible (Hopper et al., 2008). More specifically, to carry out mixed models, we used “lmer” from the “lme4” package (Bates et al., 2015). In order to compute R-squared for the models, we used “r.squaredGLMM” from MuMIn package (Barton, 2020), which takes into account the marginal R-squared (associated with fixed effects) and the conditional one (associated with fixed effects plus random effects). For each model, we reported the marginal R-squared. The p-value was also calculated using the “lmerTest” package (Kuznetsova et al., 2017).

#### EEG Statistical Analyses

To analyze oscillatory activity we implemented a two-level statistical approach. The first-level data analyses were carried out using a cluster-based procedure implemented in Fieldtrip, while in the second level of data analyses we performed mixed-effect models using R. More in detail, in the first-level analyses we decided to detect condition differences by employing an unbiased approach, testing for statistical effects across all electrode sites while controlling for multiple comparisons. Hence, we applied a whole-scalp cluster-based permutation analysis (Groppe et al., 2011) to identify illusion effects by comparing Synchronous vs. Asynchronous conditions. Specifically, a two-tailed paired *t*-test was performed for each electrode, and the cluster statistic was defined as the sum of the t-values of all spatially adjacent electrodes exceeding a critical value corresponding to an alpha level of 0.05, and a minimal cluster size of two (Maris and Oostenveld, 2007; Kayser et al., 2015). The cluster statistic was compared with the maximum cluster statistic of 1,000 random permutations, based on an overall *p*-value of 0.05. In the second-level analyses, the significant electrodes were grouped in clusters, defining three bilateral brain areas and we conducted mixed-effect model analyses on log-alpha power. The logarithmic transformation of the alpha power was used in order to improve the normality of the power distribution (Oberman et al., 2005; Lenggenhager et al., 2011).

## Results

### Cognitive Assessment

**Figure 2:**
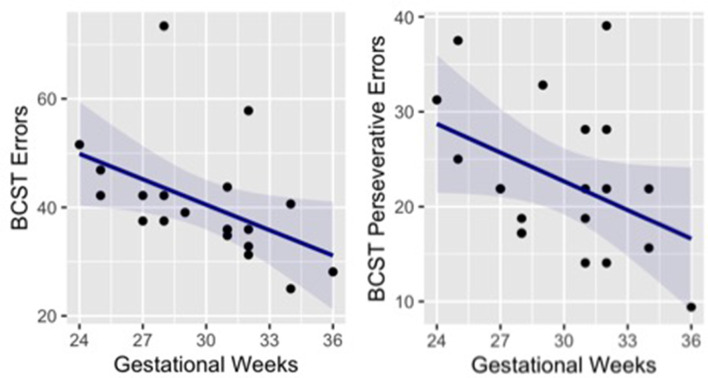
Correlation between preterm children’s gestational age and performance at the BCST (number of errors and number of perseverative errors). BCST, Berg Card Sorting Test.

Paired *t*-tests were used to test for group differences in different cognitive tests that were selected to evaluate general cognitive abilities. No significant group difference was found for performance at the CPM (*t* = −0.11, *p* = 0.915, Cohen’s *d* = −0.04), Digit Span forward (*t* = 1.31, *p* = 0.201, Cohen’s *d* = 0.43) and backwards (*t* = 1.49, *p* = 0.158, Cohen’s *d* = 0.48). Likewise, the two groups showed no difference in attentional skills, as measured by the three components of ANT: Alerting (*t* = −0.08, *p* = 0.939, Cohen’s *d* = −0.03), Orienting (*t* = −1.20, *p* = 0.241, Cohen’s *d* = −0.43) and Executive control (*t* = −0.76, *p* = 0.452, Cohen’s *d* = −0.25). However, in a more complex task that assesses cognitive flexibility as a core executive function (BCST) a significant difference between groups emerged. The percentage of errors in the group of preterm children was significantly higher than in the control group (*t* = −4.40, *p* < 0.001, Cohen’s *d* = −1.42). In particular, preterm participants made more perseverative errors than full-term participants (*t* = −3.56, *p* = 0.001, Cohen’s *d* = −1.15), while no significant difference emerged between groups in respect of non perseverative errors (*t* = −1.47, *p* = 0.152; Cohen’s *d* = −0.50; Table 2). It is important to note that non-perseverative errors are common after a rule change as a new association must be discovered using trial and error *via* feedback received after each card is sorted; however, perseverative errors identify impaired cognitive flexibility (Fox et al., 2013). Interestingly, correlations between gestational weeks and the performance at the BCST indicate that children born at lower gestational age showed higher number of errors (*r* = −0.47, *p* = 0.035) and perseverative errors (*r* = −0.42, *p* = 0.067; Figure 2).

**Table 2:** Descriptives and tests for group differences for each cognitive test.

	Preterm children	Full-term children	Test for group differences
CPM (Z scores)	0.85 (0.66)	0.87 (0.71)	*t* = −0.11 *p* = 0.915
Digit span forward (Z scores)	−0.28 (0.78)	0.10 (1.01)	*t* = 1.31 *p* = 0.201
Digit span backwards (Z scores)	0.28 (0.70)	0.69 (1.00)	*t* = 1.49 *p* = 0.158
ANT	Alerting: 30.49 (50.62) Orienting: 39.23 (39.23) Conflict: 53.38 (61.96)	Alerting: 29.30 (41.04) Orienting: 15.51 (63.84) Conflict: 38.73 (51.84)	Alerting: *t* = −0.08 *p* = 0.939 Orienting: *t* = −1.20 *p* = 0.241 Conflict: *t* = −0.76 *p* = 0.452
BCST	Errors: 40.72% (10.81) Perseverative Errors: 22.81% (7.94) Non Perseverative Errors: 15.55% (5.68)	Errors: 26.86% (8.35) Perseverative Errors: 14.56% (6.14) Non Perseverative Errors: 12.30% (7.44)	Errors: *t* = −4.40, *p* <0.001 Perseverative Errors: *t* = −3.56, *p* <0.001 Non Perseverative Errors: *t* = −1.47, *p* = 0.152

### Parent-Report Questionnaires

**Figure 3:**
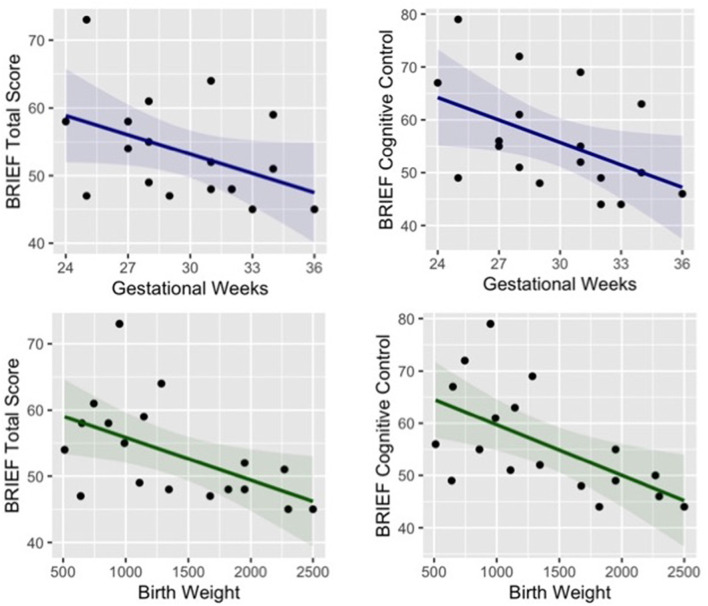
Correlation between preterm children’s gestational age (blue) and birth weight (green) and their parent-reported difficulties in executive functions (total score and subscale of cognitive control).

Paired *t*-tests were used to test for group differences in different parent-report questionnaires that were selected to evaluate children’s cognitive, emotional and social functioning in everyday activities. No significant group difference was found in any subscale of the Strengths and Difficulties Questionnaire (SDQ; Marzocchi et al., 2002); the Emotion Regulation Checklist (ERC; Shields and Cicchetti, 1997; Molina et al., 2014); and the Temperament in Middle Childhood Questionnaire (TMCQ; Simonds and Rothbart, 2004). Behavioral Rating Inventory of Executive Function (BRIEF; Gioia et al., 2000), which investigate cognitive, behavioral, and emotional executive functioning, showed a significant difference between groups in the total score (*t* = −2.07, *p* = 0.046, Cohen’s *d* = −0.71), and in particular in the subscale of cognitive control (*t* = −2.75, *p* = 0.010 Cohen’s *d* = −0.93). In order to deeper explore the risk for difficulties in executive functions in preterm children, we ran correlational analyses between the subscales of the BRIEF and neonatal information of the preterm group (gestational weeks and birth weight). The results revealed that parent-reported difficulties in executive functions are related to both lower gestational age (*r* = −0.43, *p* = 0.072) and birth weight (*r* = −0.54, *p* = 0.021) and more specifically cognitive aspects of executive function are related to lower gestational age (*r* = −0.48 *p* = 0.044) and lower birth weight (*r* = −0.60 *p* = 0.009), see Figure 3.

**Figure 4:**
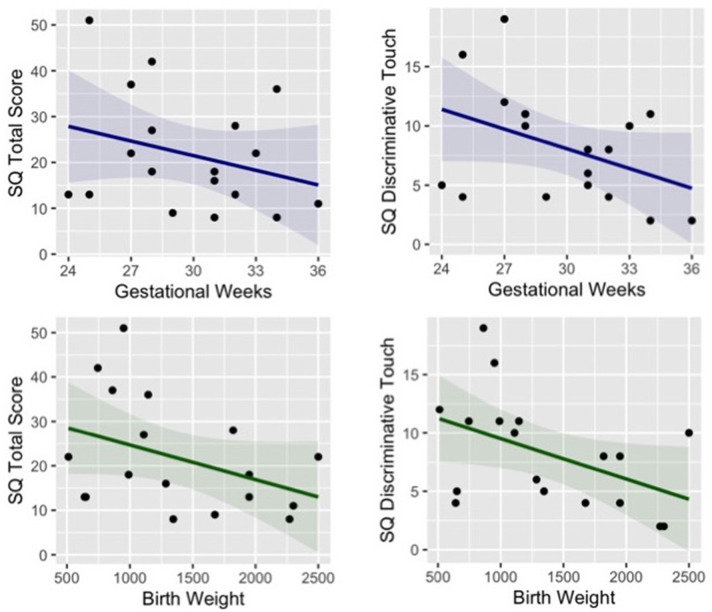
Correlation between preterm children’s gestational age (blue) and birth weight (green) and their parent-reported sensory difficulties (total score and subscale of discriminative touch).

Finally, the sensory questionnaire revealed a significant difference between groups in the total score (*t* = −2.14, *p* = 0.041, Cohen’s *d* = −0.71) and in particular in the subscale Discriminative Touch (*t* = −2.94, *p* = 0.007, Cohen’s *d* = −0.98). Moreover, a trend for significant correlation between somatosensory difficulties (total score and subscale discriminative touch) and neonatal information (gestational weeks and birth weight) emerged in preterm children, showing that parent-reported sensory difficulties appear to be negatively related to both gestational age (Total score *r* = −0.40, *p* = 0.240; Discriminative Touch *r* = −0.41, *p* = 0.093) and birth weight (Total score *r* = −0.29, *p* = 0.110; Discriminative Touch *r* = −0.46, *p* = 0.052), see Figure 4.

### Behavioral Results: Proprioceptive Drift

At first we ran some preliminary analyses on the baseline estimation error of hand position to the end of controlling whether the two groups were comparable in their proprioceptive ability to localize their own hand without any visuotactile stimulation. In particular, estimation error was calculated as the difference between the mean pointing response before visual-tactile stimulation (baseline) and the actual hand position, in both groups of full-term and preterm children. The results revealed that all children were accurate in the localization of their own hand as suggested by simple *t*-tests comparing the baseline pointing with the real position of the hand (zero). Moreover, no significant difference emerged between groups (Table 3).

**Table 3:** Simple *t*-test comparing baseline pointing score with the real position of the hand (baseline estimation error in cm) and independent *t*-test testing the difference between groups.

	Preterm children	Full-term children
Baseline estimation error (cm)	−0.37 (3.45)	−0.93 (2.88)
Simple *t*-test (null level)	*t* = −0.49, *p* = 0.629	*t* = −1.42, *p* = 0.174
Independent *t*-test	*t* = −0.56, *p* = 0.576, Cohen’s *d* = −0.18	

Then, we analyzed the proprioceptive drift, which was calculated as the difference between the perceived position of the hand after the visuotactile stimulation and the baseline location of the participants’ hand. We used a mixed-effect model approach testing five nested mixed-effects models. In each model, proprioceptive drift was the dependent variable. The null model (Model 0) included only the random effect of Participants; the first (Model 1) included the experimental Condition (two levels; synchronous visuotactile stimulation vs. asynchronous visuotactile stimulation) as fixed factor and Participants as a random factor. Moreover, we were interested in investigating possible differences between preterm and full-term children; therefore, we tested two additional models including the Group (two levels; preterm vs. full-term children) as a fixed factor (Model 2) and the interaction effect (Model 3). Finally, we wanted to control whether developmental changes may influence the effects of the RHI, therefore we tested an additional model including age in months as a fixed factor (Model 4; Table 4).

**Table 4:** Comparison between models predicting proprioceptive drift.

Tested models	Variables	AIC	Delta AIC	Marginal *R*^2^	*χ* ^2^	*p*
Model 0	Random effect of participants	419.01				
Model 1	+ Condition	415.94	3.19	0.016	5.06	0.024
Model 2	+ Group	415.86	2.11	0.058	2.09	0.149
Model 3	+ Condition × Group	415.78	1.52	0.064	2.08	0.149
Model 4	+ Age	417.70	−6.73	0.064	0.08	0.784

*Note that smaller values of AIC indicate better fitting models*.

**Figure 5:**
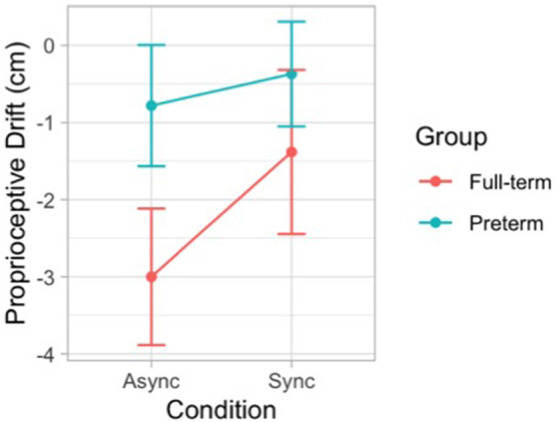
Error bar graph of the mean proprioceptive drift following synchronous and asynchronous visuotactile stimulation in full-term (red) and preterm (blue) children. The graph shows the interaction effect between visual-tactile condition and group.

The likelihood ratio test showed that Model 3 was the best at predicting the collected data and included the effect of visual-tactile Condition, the effect of Group, and the interaction effect Condition × Group. We selected this model, even though it did not reach statistical significance (*p* = 0.149) because it was associated with a smaller AIC indicating a better fit of the collected data and it increased the percentage of explained variance (6%). The main effect of Condition emerged as significant (*B* = 1.62, *SE* = 0.61, *t* = 2.63, *p* = 0.012). Moreover, the model showed a trend for the main effect of Group (*B* = 2.22, *SE* = 1.21, *t* = 1.84, *p* = 0.073) and an interaction effect Condition × Group (*B* = −1.21, *SE* = 0.85, *t* = −1.42, *p* = 0.163), although statistical significance was not reached. These results indicate that when the touch applied on the participant’s actual hand was asynchronous compared to the touch applied on the rubber hand, children showed a larger proprioceptive drift away from the real hand. This effect shows a different modulation in the two groups of participants (Figure 5).

### Behavioral Results: Embodiment

Then, we analyzed the subjective experience of feeling a sense of embodiment over the rubber hand after the manipulation of multisensory signals. A set of five nested mixed-effect models was tested including the same factors used to analyze proprioceptive drift (Table 5).

**Table 5:** Comparison between models predicting the subjective experience of embodiment over the rubber hand.

Tested models	Variables	AIC	Delta AIC	Marginal *R*^2^	*χ* ^2^	*p*
Model 0	Random effect of participants	343.95				
Model 1	+ Condition	317.22	25.88	0.162	28.73	<0.001
Model 2	+ Group	312.60	4.94	0.261	6.62	0.010
Model 3	+ Condition × Group	314.55	−1.39	0.260	0.05	0.820
Model 4	+ Age	316.39	−8.25	0.258	0.15	0.696

*Note that smaller values of AIC indicate better fitting models*.

**Figure 6:**
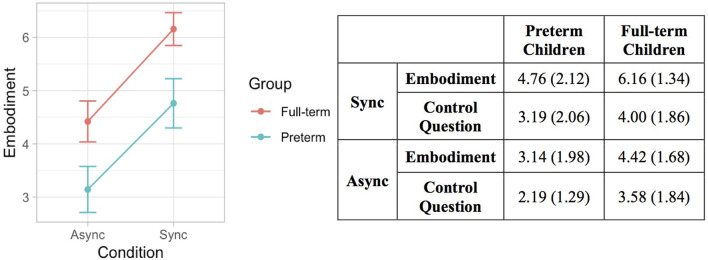
Error bar graph of the mean embodiment score following synchronous and asynchronous visuotactile stimulation in full-term (red) and preterm (blue) children. The graph shows the main effects of condition and group.

The likelihood ratio test showed that Model 2 was the best at predicting the collected data and included factors Condition and Group. The model explained 26% of the variance (*p* = 0.010). The main effects of Condition (*B* = 1.68, *SE* = 0.26, *t* = 6.40 *p* < 0.001) and Group (*B* = −1.34, *SE* = 0.51, *t* = −2.62, *p* = 0.013) emerged. These results indicate that children felt a stronger sense of ownership over the rubber hand when the touch was delivered synchronously on the participant’s real hand and the rubber hand. This finding is in line with qualitative observations of children’s behavior during the induction of the illusion, as many of them exhibited surprise and made spontaneous verbal comments about feeling the rubber hand as part of their own body. Moreover, preterm children reported lower scores in response to the ownership question in both conditions of the RHI. Notably, the same pattern of scores was shown for the control question with the important difference that in all experimental conditions the mean values were above 4, which corresponded to the middle value of the scale indicating uncertainty whether or not the illusory effect was applied (Figure 6).

### EEG Results

**Figure 7:**
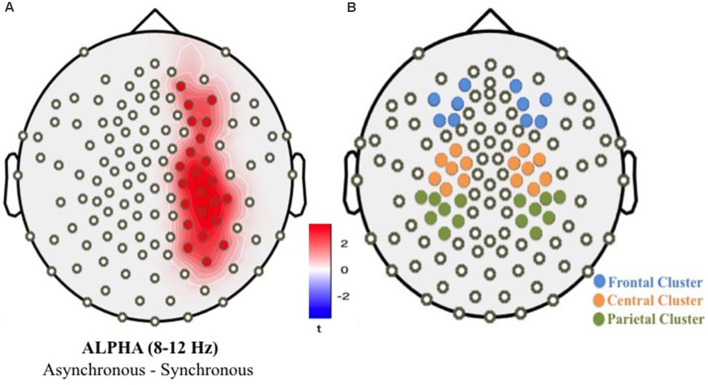
Cluster of significant electrodes resulting from the first-level permutation analyses contrasting asynchronous vs. synchronous conditions **(A)**; bilateral areas of interest considered in second-level analyses **(B)**.

In the first-level oscillatory analyses, we applied an exploratory analysis by testing the difference between experimental conditions (asynchronous vs. synchronous) considering the full sample of children (full-term and preterm). This first-level analysis was performed to individuate the electrodes showing any condition-related significant modulation. We used a cluster-permutation procedure to control for multiple comparisons (detailed parameters: 1,000 iterations, two-sided *t*-test at *p* < 0.05 on the clustered data, requiring a cluster size of at least 2 significant neighbors). The condition contrast applied to the power of oscillatory activity revealed a significant cluster of 22 electrodes over the right frontal, central and parietal areas, where alpha power (8–12 Hz) was lower in the synchronous condition compared to the asynchronous condition (Figure 7A). A second-level, confirmatory analysis was then performed, with the specific aim of assessing the presence of group-level significant differences. For this purpose, we selected three bilateral clusters of electrodes, covering frontal, central, and posterior parietal brain areas (Figure 7B). These clusters were identified according to both the exploratory analysis and previous literature (Evans and Blanke, 2013; Rao and Kayser, 2017).

We then computed and averaged the mean power spectrum density of all the electrodes within each cluster, separately for the full-term and preterm groups of children. In this way, we obtained a single, averaged power density value per cluster and participant and entered these values into a mixed model approach. A set of seven nested mixed-effect models was tested considering the mean alpha power as our dependent variable. The null model (Model 0) included only the random effect of Participants; the first (Model 1) included Condition (two levels; synchronous visuotactile stimulation vs. asynchronous visuotactile stimulation) as fixed factor and Participants as random factor. Moreover, we were interested in investigating possible differences between preterm and full-term children; therefore, we tested two additional models including the Group (two levels; preterm vs. full-term children) as a fixed factor (Model 2) and the interaction effect (Model 3). Finally, we tested three additional models including Scalp area (3 levels; frontal vs. central vs. parietal, Model 4), Laterality (2 levels; right vs. left, Model 5), and their interaction (Model 6) as fixed factors (Model 5; Table 6).

**Table 6:** Comparison between models predicting oscillatory activity in the alpha frequency band.

Tested models	Variables	AIC	Delta AIC	Marginal *R*^2^	*χ* ^2^	*p*
Model 0	Random effect of participants	−103.48				
Model 1	+ Condition	−138.77	28.91	0.022	37.29	<0.001
Model 2	+ Group	−137.49	−4.23	0.035	0.73	0.394
Model 3	+ Condition × Group	−139.76	−2.74	0.037	4.27	0.039
Model 4	+ Scalp Area	−148.52	−3.60	0.044	12.76	0.002
Model 5	+ Laterality	−166.77	11.61	0.055	20.25	<0.001
Model 6	+ Scalp Area × Laterality	−163.34	−13.04	0.055	0.58	0.750

*Note that smaller values of AIC indicate better fitting models*.

**Figure 8:**
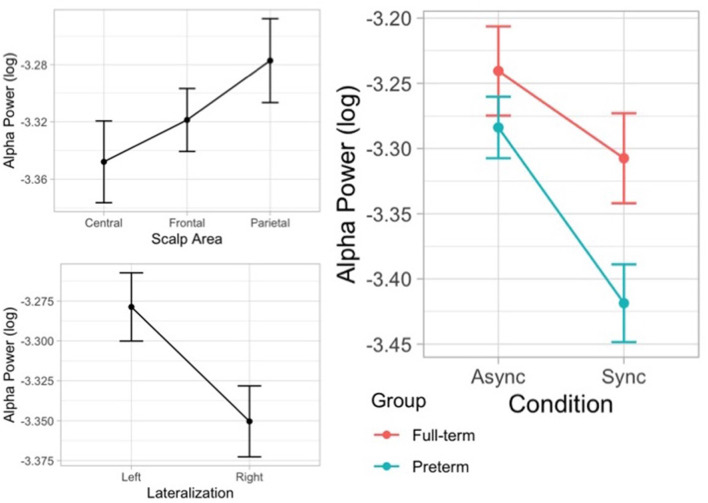
Error bar graphs of the mean alpha power showing the main effects of scalp area, lateralization, and the interaction effect between visual-tactile condition (asynchronous vs. synchronous) and group (full-term vs. preterm).

The likelihood ratio test showed that Model 5 was the best model at predicting the collected data and included the factors Condition, Group, Condition × Group, Scalp area, and Laterality. The model explained 5% of the variance (*p* < 0.001). The main effect of Condition (*B* = −0.07, *SE* = 0.02, *t* = 2.91 *p* = 0.004) and Laterality (*B* = 0.07, *SE* = 0.02, *t* = 4.53, *p* < 0.001) emerged. These results indicate that children displayed higher alpha desynchronization, as reflected by a decreased power, on the contralateral scalp side of stimulation and that they show a greater alpha suppression when the touch was delivered synchronously on the participant’s real hand and on the rubber hand compared to the asynchronous condition. Moreover, alpha power density varied across regions on the scalp showing a higher alpha power over parietal area (*B* = 0.07, *SE* = 0.02, *t* = 3.64 *p* < 0.001). Finally, an interaction effect between visual-tactile condition and group emerged (*B* = −0.07, *SE* = 0.03, *t* = −2.14 *p* = 0.033), suggesting that preterm children showed a greater suppression of alpha oscillatory activity during the illusion (Figure 8).

### Correlation Between Behavioral Measures and Alpha Power

**Figure 9:**
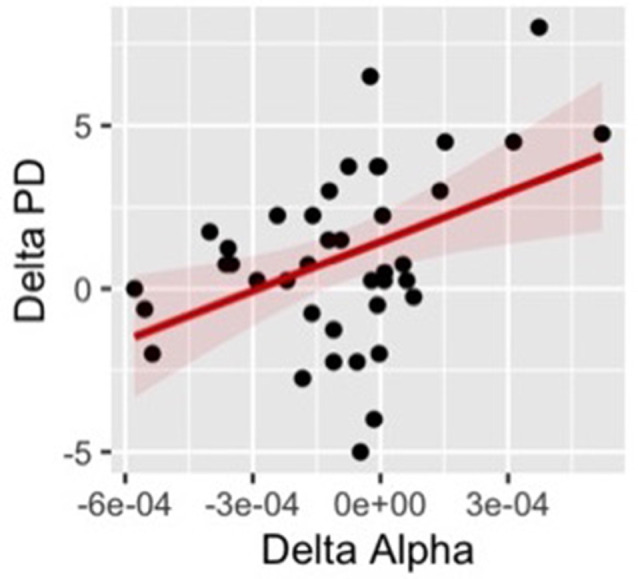
Positive correlation between changes in proprioceptive drift and alpha power.

Finally, we ran correlational analyses searching for a possible relationship between changes in EEG activity and changes of self-location at the individual level. Specifically, we calculated the difference (delta score) between the synchronous and asynchronous conditions in both proprioceptive drift and alpha power and we computed the correlation for these measures separately for each scalp area. The results revealed a positive correlation between the changes in proprioceptive drift and alpha power (*r* = 0.43 *p* = 0.006) over the right frontal cluster (Figure 9). The positive correlation indicates that participants with larger changes in the perceived position of their hand between the synchronous and asynchronous visual-tactile stimulation, showed larger modulation of alpha oscillatory activity in electrodes over the right frontal area.

## Discussion

During childhood, the body undergoes numerous changes, pointing to the need of constantly monitoring and integrating bodily signals in order to update the representation of one’s own body, which mediates all interactions with the external physical and social world and represents a foundation for the development of individual psychological identity as separate from the others. Specifically, bodily self-awareness relies on the sense of body ownership, which refers to the feeling that our body belongs to ourselves, and the localization of our body in the environment, based on the experience that our body occupies a given location in space (Serino et al., 2013). The RHI paradigm allows investigating the flexibility of the sense of body ownership, as a result of multisensory processes that integrate the tactile input felt on the actual hand with the visual feedback seen on the rubber hand. The main correlates of the RHI are the experience of feeling a rubber hand as part of one’s own body (subjective embodiment) and the misallocation of the own hand towards the rubber hand (proprioceptive drift). A primary aim of this study was to explore sensitivity to the RHI in childhood, focusing on the classical behavioral measures as well as on the oscillatory activity during synchronous and asynchronous visual-tactile stimulation. Moreover, we were interested in investigating whether the deprivation of parent-infant bodily contact in the neonatal period, such as in the case of preterm birth, bears long-term negative consequences for the development of bodily self-awareness. Thus, a second purpose of the present study was to explore whether preterm children present less susceptibility to the RHI compared to full-term children, which may indicate atypical integration of multisensory bodily signals.

In order to achieve our objectives, we presented full-term and preterm school-age children with the RHI. The two groups of participants have been shown not to differ for abstract reasoning, as evaluated in performance at the Raven’s Colored Progressive Matrices (CPM; Raven, 1958), short-term (forward digit span) and working memory (backward digit span; BVN 5–11; Bisiacchi et al., 2005) and attention skills, as measured by the Attention Network Task (ANT; Rueda et al., 2004). This general cognitive assessment was designed in order to ensure comparable cognitive levels between the two groups. Similarly, parent-report questionnaires indicated no difference in behavioral and emotional difficulties (SDQ; Marzocchi et al., 2002), emotion regulation (ERC; Shields and Cicchetti, 1997; Molina et al., 2014), and temperament (TMCQ; Simonds and Rothbart, 2004) between the two groups of children. However, in line with previous studies (Mulder et al., 2009), preterm children were found to have some difficulties in executive functioning as indicated by a lower performance at the Berg Card Sorting Test (BCST; Berg, 1948). In particular, preterm children showed a higher number of perseverative errors, whereas no difference emerged in the non-preservative errors, indicating that preterm children may present a specific impairment in inhibiting a prevalent response when they are challenged with a complex task. In line with this finding, parents of preterm children reported more difficulties in cognitive control during everyday activities compared to parents of full-term children, as measured by the Behavioral Rating Inventory of Executive Function (BRIEF; Gioia et al., 2000). Executive functions, which refer to the self-regulation processes involved in emotion, cognition, and goal-directed behavior (Diamond, 2013) are frequently compromised in preterm children, especially in those with more extreme prematurity (Taylor and Clark, 2016). Indeed, in the present study, correlational analyses revealed that children born at lower gestational age showed more difficulties in executive function, as measured by children’s performance at the BCST and parents’ reported scores in the subscale of cognitive control of the BRIEF. Furthermore, the sensory questionnaire revealed a significant difference between groups in the total score and in the subscale of Discriminative Touch. Notably, a trend for a significant correlation between somatosensory difficulties and neonatal information (gestational weeks and birth weight) emerged in preterm children, showing that parent-reported sensory difficulties are prevalent in children born at lower gestational age and birth weight. These results are in line with previous findings which suggest that sensory difficulties related to preterm birth persist during childhood with important implications for neurocognitive and behavioral outcomes (Bröring et al., 2017; Ryckman et al., 2017).

The behavioral measures obtained from the RHI task are in line with previous studies (Cowie et al., 2013, 2016; Nava et al., 2017; Filippetti and Crucinelli, 2019), revealing that school-age children are able to modulate their body representation based on the integration of visual-tactile information, as reflected by the higher subjective feeling of embodiment over the rubber hand in the synchronous compared to the asynchronous experimental condition. The illusion was often very vivid for the children who made spontaneous verbal comments and exhibited reactions of excitement or surprise. The visual-tactile synchrony, meaning the spatial and temporal consistency between the visual and the tactile information is a major factor underpinning the RHI that has been consistently reported in both adult and children studies (e.g., Botvinick and Cohen, 1998; Ehrsson et al., 2004; Bekrater-Bodmann et al., 2014; Cowie et al., 2016). The principles of spatio-temporal congruence constrain the selection of the multisensory signals that are to be combined. In the synchronous condition, participants integrate the tactile sensation felt on their actual hand with the visual information on the rubber hand and adjust their body representation in order to maintain a unitary representation of the self, resulting in an illusory sense of ownership over the rubber hand. By contrast, when the touch is administered on different fingers (spatial discrepancy) or asynchronously between the real hand and the rubber hand (temporal discrepancy), the illusion is abolished (Botvinick and Cohen, 1998; Kammers et al., 2009). More specifically, a strong sensation of the RHI occurs when the temporal discrepancy is 300 ms or less, while it decreases as the delay lengthens (Bekrater-Bodmann et al., 2014; Shimada et al., 2014). Similarly, the distance between the real hand and the rubber hand has proven to modulate the strength of the illusion (Erro et al., 2020), with a significant decrease of illusory effect after a distance of 30 cm (Lloyd, 2007). Notably, developmental studies investigating multisensory processes suggest that the spatial distance and the temporal window within which multisensory stimuli are likely to be integrated into a unitary experience narrows over childhood (Greenfield et al., 2017), suggesting that children might partially perceive the RHI even during asynchronous conditions. Future investigation should investigate this possibility by systematically manipulating the length of temporal delay and spatial distance between visual and tactile stimulations in an RHI paradigm.

We also found a shift of the children’s proprioceptive perceived position of the real hand relatively closer to the rubber hand when the visual-tactile stimulation was synchronous compared to the asynchronous condition. It is important to notice that overall, values obtained from the pointing task indicate that children tended to localize their own hand away from the rubber hand, although this effect was reduced for the synchronous condition. One may find this result inconsistent with previous findings in developmental populations, which on the contrary reported a bias towards the body midline even without any stroking (Cowie et al., 2013, 2016; Nava et al., 2017; Filippetti and Crucinelli, 2019). This unexpected negative bias is not easy to interpret, however, given the fact that it appears to spread to all experimental conditions and in both groups of participants, it is still relevant to investigate whether children modulate the perceived hand position based on the integration of visual-tactile stimulation. Indeed, our results revealed a main effect of synchrony on proprioceptive drift resampling the classical effect that participants tend to localize their own hand closer to the rubber hand during the synchronous compared to the asynchronous stroking (Botvinick and Cohen, 1998). However, it has to be taken into account that developmental results regarding the proprioceptive drift in children are complex and they do not agree on the relationship with age. Indeed, all RHI studies consistently reported a stable and early developing subjective experience of ownership over the rubber hand in children from 4 to 13 years (Cowie et al., 2013, 2016; Nava et al., 2017; Filippetti and Crucinelli, 2019), but they showed different results concerning the recalibration of hand position. While in some studies young children showed a greater proprioceptive shift towards the rubber hand compared to older children and adults, suggesting a strong reliance on visual information (Cowie et al., 2016), other findings pointed to a different developmental pathway in the ability to localize one’s own hand (Nava et al., 2017), yet another study failed to find any effect of recalibration of hand position towards the rubber hand (Filippetti and Crucinelli, 2019). Therefore, developmental research up to now seems to provide evidence in support of adults studies that found a dissociation between subjective (embodiment questionnaire) and behavioral (proprioceptive drift) measures of the RHI (Rohde et al., 2011; Abdulkarim and Ehrsson, 2016), raising the question about how multisensory processes underpinning different components within body ownership develop across childhood (Cowie et al., 2016; Filippetti and Crucinelli, 2019). More specifically, the subjective feeling of an embodiment is consistently sensitive to visual-tactile synchrony and it has been shown not to depend on changes in the hand position sense (Abdulkarim and Ehrsson, 2016). This effect emerges early in life as children of all ages appear to experience a sense of ownership over the rubber hand in much the same way as adults (Cowie et al., 2016). By contrast, proprioceptive drift relies on visuo-proprioceptive integration and it seems to be inhibited by asynchronous stroking (Rohde et al., 2011). The developmental trajectory of the effect of the RHI on proprioceptive hand position across childhood is still unknown, as different studies reported inconsistent results, possibly due to the variation in experimental paradigms. Indeed, significant differences in methodological approaches and conceptualization make it difficult to integrate findings across different studies, suggesting the need for a more coherent body of literature for developmental RHI studies (Lee et al., 2021).

In particular, a substantial lack of understanding concerns the neural signatures of the multisensory mechanisms underlying the development of body ownership. To our knowledge, no studies have yet examined neural responses during the RHI in the developmental population. Thus, an essential aim of this study was to fill this research gap investigating children’s oscillatory activity in order to provide some insight into the multisensory processes underpinning the RHI. EEG oscillatory activity results suggest that in the synchronous condition all children showed a suppression of alpha frequency bands over the right frontal, central, and parietal scalp regions in respect to the asynchronous condition, pointing to an increased involvement of the brain network supporting multisensory body-related processing. More specifically, alpha band oscillations over central regions have been linked to sensorimotor processing (Pineda, 2005). Central alpha suppression, indicated by a decrease of spectrum power, is caused by neuronal desynchronization and reflects increased cortical activation in sensorimotor and premotor cortices (Oakes et al., 2004). Central alpha suppression has been linked to somatosensory stimulation and observation of touch of another person (Pfurtscheller, 1981; Cheyne et al., 2003) as well as action execution, observation, and imagery (Gastaut, 1952; Pfurtscheller and Neuper, 1997). Stronger activations in the synchronous visuotactile condition could be related to mechanisms of multisensory body processing, suggesting a greater engagement of cortical areas associated with visuotactile body-related integration. In support of this interpretation, functional neuroimaging studies supported the activation of a diffuse network of brain areas during the illusory self-attribution of a rubber hand (Ehrsson et al., 2004, 2005; Tsakiris et al., 2007; Tsakiris, 2010; Blanke, 2012). Different brain regions including premotor cortex (Ehrsson et al., 2004), sensorimotor cortex, intraparietal sulcus (Kammers et al., 2009), temporoparietal junction (Tsakiris et al., 2008), and insula (Tsakiris et al., 2007; Baier and Karnath, 2008) work in concert integrating vestibular, visual, and somatosensory signals providing a foundation for self-identification and self-location processes (Blanke, 2012; Limanowski and Blankenburg, 2015).

Another important finding of the present study is that a positive correlation emerged between modulation of alpha band power in the right frontal site and the shift of proprioceptive position of the hand after the illusory visuotactile stimulation. In line with previous results from adult research (Lenggenhager et al., 2011), this positive correlation links frontal oscillatory activity with self-location processes suggesting that participants with a stronger illusory misallocation of their own hand showed decreased frontal activation, as reflected by an increased alpha power. Indeed, activity in the medial prefrontal cortex has been associated with various aspects of the self, including linguistic self-reference (Esslen et al., 2008), memory for self-relevant personality characteristics (Macrae et al., 2004), and self-other discrimination (Heatherton et al., 2006). Notably, neurological patients with impaired frontal processing presented specific alterations of self-location and self-identification (Heydrich et al., 2010; Lopez et al., 2010). Therefore, it is reasonable that frontal activation may reflect the robustness of self-representation and consequently different susceptibility to body illusions. Accordingly, our results evidenced that, contrasting the synchronous vs. the asynchronous visual-tactile conditions, increased oscillatory power in a distributed network where frontal areas have a central role is associated with a stronger illusory bias in self-location. This result provides important insight into possible individual differences that may mediate the effect of the illusion. Notably, interoceptive predictive models provided enlightening evidence in this direction, suggesting that individuals who strongly rely on internal bodily signals presented a less malleable sense of body ownership. Specifically, individual differences in interoceptive sensitivity, measured as the ability to accurately detect the own heartbeat, has been shown to predict proprioceptive drift during the RHI (Tsakiris et al., 2011). A possible interpretation is based on the fact that during the RHI there is a conflict between the proprioceptive input about the hand position and the visual information about the location of the rubber hand and the tactile stimulation, thus the brain has to resolve uncertainty by weighting multisensory inputs in order to select some sources of information and down-regulate other conflicting somatosensory cues (Zeller et al., 2016). In order to minimize predictor errors, high-level body representation is also integrated and updated, resulting in a modulation of the sense of body ownership when the illusion occurs (Limanowski and Blankenburg, 2015). In this perspective, our results indicate that variation of activation of frontal areas, which is typically observed during self-referential processes, correlates with modulation of self-localization, possibly suggesting that participants who are able to inhibit a self-anchored representation of their body, are more likely to recalibrate their perceived hand position during the RHI.

Concerning the difference between preterm and full-term children, the results of the present study suggest that both groups of children showed sensitivity to the RHI, as they reported significantly higher scores of embodiment during synchronous visual-tactile stimulation compared to the asynchronous condition. Therefore, preterm children appear to be able to modulate the representation of their body on the basis of multisensory integration processes, as typically developmental children do. However, our results also indicate a main significant effect of the group, suggesting that overall preterm children reported lower scores of subjective embodiment over the rubber hand. A possible interpretation of this result is that preterm children may be more anchored on a stable representation of their body, thus showing more difficulty in modulating their bodily boundaries in order to include external objects as part of their own body, irrespective of the available multisensory information. This speculation is further supported by the data from the proprioceptive drift, which showed a different modulation of the proprioceptive perceived position in preterm and full-term children, although statistical significance was not reached. In particular, full-term children exhibited a greater recalibration of self-localization due to different visuotactile stimulation, whereas preterm children showed a more persistent response close to the initial proprioceptive perceived position. The reduced embodiment of the rubber hand and the accurate localization of the hidden hand could indicate a bias towards proprioceptive processing. It is possible that preterm children presented an unusual strong reliance on proprioception and atypical multisensory integration of other body-related cues. Notably, similar atypicalities in behavioral measures of the RHI have been found in children with autism spectrum disorder (ASD) who displayed a delayed susceptibility to the illusion after 6 min of stimulation and difficulty in differentiating their subjective experiences between asynchronous and synchronous stimulations (Cascio et al., 2012). Compared to children with typical development, both groups of autistic and preterm children showed a stronger tendency to focus on proprioceptive signals ignoring competing information from other sensory modalities, resulting in less susceptibility to the conflicting visual and tactile input during the induction of the RHI. By systematically varying the brushing period from 2 to 6 min, future studies could investigate the possibility that prolonged visuotactile stimulation may eventually lead to a remapping of the body representation in preterm children, as evidenced in children with ASD. Considering the neural measures, preterm children revealed a different modulation of alpha oscillatory activity compared to full-term children during the RHI. In particular, they showed a greater alpha suppression which may reflect a greater effort in integrating multisensory bodily signals. Taken together, our findings might suggest that although preterm children showed sensitivity to different visual-tactile stimulation suggesting the ability to integrate multisensory information, they also appeared to be more anchored to their own body and to place a greater reliance on internal proprioceptive information rather than external sensory cues. Indeed, they seem to be less likely to perceive the rubber hand as part of their own body irrespective of the visual-tactile stimulation, possibly indicating a more rigid representation of their own body.

Accurate integration of visual, tactile, and proprioceptive input underpins the sense of bodily self with important implications for identifying, differentiating, and comparing oneself with others (Meltzoff, 2007; Tsakiris, 2016). The malleability of the sense of body ownership allows a partial overlap of our body and those of others (Maister et al., 2015), as reflected by a shared representation of the self and the other in the brain, which may underpin the basis of social understanding and social connection (Brozzoli et al., 2013; Courtney and Meyer, 2020). Indeed, the multisensory representation of the body not only guides self-awareness and sensorimotor development, but also provides an interpretative framework for understanding the actions, goals, and psychological states of others, critically influencing the ability to successfully engage in social interactions (Ropar et al., 2018). It has been shown that the embodiment of a different body to one’s own with respect to gender, age, or race changes the representation of one’s own body through a process of self-other association that first takes place in the bodily domain (Maister et al., 2015). In tune, the perceived physical similarity between the self and another outgroup person extends to the socio-cognitive domain, resulting in a reduction of implicit biases against outgroup members and modulation of social cognition processing (Paladino et al., 2010; Farmer et al., 2014). Thus atypical body-related multisensory integration could affect the development of body ownership and the malleability of one’s own bodily representation, impacting higher-order social and cognitive processes, including the understanding of others’ actions and emotions (Ropar et al., 2018). In support of this, a recent study showed that lacking bodily contact in the first weeks of life due to prenatal birth affected mother-infant synchrony (Yaniv et al., 2021). However, if additional skin-to-skin mother-newborn contact was provided, an increased mother-child synchrony was observed across development impacting the brain’s capacity to empathize with others in adulthood (Yaniv et al., 2021). These findings support that early sensory experiences shape the representation of one’s own body as a point of reference for interactions with the external physical and social environment with cascading effects on socio-emotional and cognitive development. Thus, studying the integration of different sensory signals in the context of the RHI may have a crucial relevance for better understanding typical and atypical developmental trajectories, sharing light on the developmental processes of the acquisition of a sense of body ownership and differentiation of the self from the others as precursors to more complex social behaviors. Specifically, the results of the present study point to a possible less malleable representation of the body in preterm children that should be better investigated in light of neurobiological vulnerability and early exposure to a detrimental sensory environment in NICU. This will help to assume a more comprehensive perspective on sensory, cognitive, and social development, with the potential of refining assessment methods and developing multidimensional interventions that include multisensory body-related stimulation.

In conclusion, the development of bodily-related multisensory processing and integration represents important precursors of self-awareness processes that organize sensation from different sensory channels, modulating perception of the bodily self and others. In typically developing children visual-tactile synchrony provides the basis for updating the sense of body ownership, as reflected by behavioral measures and alpha oscillatory activity. Preterm children, who are typically exposed to a detrimental sensory environment in the neonatal period, showed to be able to integrate incoming multisensory information to update the representation of their body. However, they appear to be more self-anchored, as reflected by the overall lower feeling of embodiment over the rubber hand. The findings of the present study pave the way for a multisensory approach to the investigation of social and cognitive development, focusing on the bodily self as a point of reference for the integration of sensory experiences. We believe that this line of research provides an essential contribution to better understand the processes of identification and differentiation between the self and the external environment, in both typical and atypical development.

## Data Availability Statement

The raw data supporting the conclusions of this article will be made available by the authors, without undue reservation.

## Ethics Statement

The studies involving human participants were reviewed and approved by Comitato etico della ricerca psicologica Area 17 Department of Psychology, University of Padua. Written informed consent to participate in this study was provided by the participants’ legal guardian/next of kin.

## Author Contributions

LD, TF, and GM discussed the project. LD and TF developed the hypothesis, designed the method, and prepared the materials. LD collected the data and GM supervised the data collection. LD analyzed the data and GM supervised EEG data preprocessing and oscillatory analyses. LD prepared the manuscript. All authors contributed to the article and approved the submitted version.

## Conflict of Interest

The authors declare that the research was conducted in the absence of any commercial or financial relationships that could be construed as a potential conflict of interest.

## Publisher’s Note

All claims expressed in this article are solely those of the authors and do not necessarily represent those of their affiliated organizations, or those of the publisher, the editors and the reviewers. Any product that may be evaluated in this article, or claim that may be made by its manufacturer, is not guaranteed or endorsed by the publisher.
